# Helium pencil beam commissioning and beam modeling

**DOI:** 10.1016/j.phro.2026.101046

**Published:** 2026-07-26

**Authors:** Lukas Martin, Barbara Knäusl, Peter Kuess, Dietmar Georg, Hugo Palmans, Lorenz Wolf, Nadia Gambino, Fabio Farinon, Andrej Prochazka, Mansure Schafasand, Lars Glimelius, Walter Ikegami Andersson, Daniel Simon Colomar, Antonio Carlino, Markus Stock, Hermann Fuchs

**Affiliations:** a MedAustron Ion Therapy Center, Marie Curie-Straße 5, Wiener Neustadt, A-2700, Austria; b Department of Medical Physics at the MedAustron site in Wiener Neustadt, Faculty of Medicine, Karl Landsteiner University, Marie Curie-Straße 5, Wiener Neustadt, 2700, Austria; c Department of Radiation Oncology, Medical University of Vienna, Währinger Gürtel 18-20, Vienna, A-1090, Austria; d Christian Doppler Laboratory for Image and Knowledge Driven Precision Radiation Oncology, Medical University of Vienna, Währinger Gürtel 18-20, Vienna, A-1090, Austria; e Radiotherapy and Radiation Dosimetry, National Physical Laboratory, Hampton Rd, Teddington, TW 11 0LW, United Kingdom; f RaySearch Laboratories AB, Eugeniavägen 18C, Stockholm, 113 68, Sweden

**Keywords:** Helium ion therapy, Particle therapy, Beam commissioning, Dosimetry, Monte Carlo simulation

## Abstract

**Background and Purpose:**

Helium ions combine reduced lateral scattering compared to protons and a lower fragmentation tail than carbon ions, enabling sharp dose gradients and improved normal tissue sparing. This study reports the commissioning of a scanned helium pencil beam line and validation of its corresponding beam model.

**Materials and Methods:**

Synchrotron-based helium ion beams were commissioned covering energies from 54.6 to 402.8 MeV/u. Depth–dose curves were measured and absolute dose calibration was performed. Beam optics (spot size, position, and intraspill stability) were evaluated for various spill lengths. A beam model was implemented in the RayStation treatment planning system (TPS) and validated through 2D absolute dose measurements in homogeneous and heterogeneous phantoms and 3D measurements of cubic spread-out Bragg peak fields. Gamma-index analysis and Monte Carlo (MC) simulations with GATE/Geant4 were performed for benchmarking.

**Results:**

Measured ranges agreed with MC simulations within ±0.3 mm. Spot sizes decreased with energy, independently of the spill length. Spot positions remained within ±0.5 mm and intraspill variations were ≤0.2 mm (position) and ≤5.4 % (size). TPS-predicted doses agreed within 0.1 %. For 3D validations in homogeneous phantoms, the dose differences were generally within 2 %. Median gamma pass rates exceeded 90 % for 3 %/1.5 mm and 95 % for 5 %/1.5 mm. For the heterogeneous phantom, differences were within -3.2 %.

**Conclusions:**

Stable scanned beam delivery with helium ions was established. Validation demonstrated strong agreement between measurements, TPS calculations, and MC simulations, supporting research and future clinical application.

## Highlights

• Commissioned helium ion beams from 54.6 to 402.8 MeV/u.
• Measured ranges agreed with Monte Carlo within ±0.3 millimeter across 346 energies.
• Absolute dose agreement within 0.1 percent at reference depth.
• Gamma pass rates exceeded 95 percent at 5 percent and 1.5 millimeter.
• Validated helium dose in an anthropomorphic head phantom within 3.2 percent.

## Graphical abstract

**Figure:**
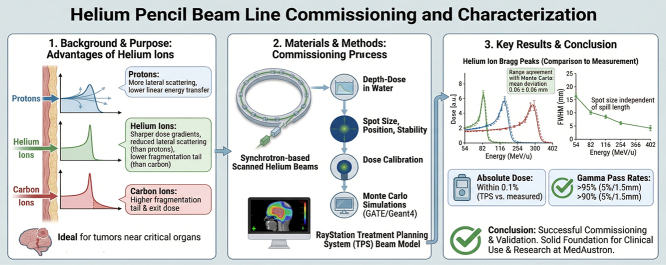

## 1 Introduction

Although protons and carbon ions have become an integral part of modern radiotherapy, other particle species were explored and clinically applied in earlier decades. At the Lawrence Berkeley National Laboratory, helium ions were used to benchmark photon therapy and other ion species using the passive scattering technique [1], [2].

Helium ions combine enhanced relative biological effectiveness (RBE), higher linear energy transfer (LET), and a sharper lateral penumbra compared to protons with a reduced fragmentation tail compared to carbon ions [3], [4], [5], [6]. These properties enable more conformal dose distributions and improved sparing of normal tissues [1], making helium ions an ideal candidate for pediatric treatments and recurrent tumors [7]. The reduced scattering also limits spot-size growth across large distances, making the air gap less critical [8], [9].

The clinical feasibility of scanned helium ion therapy was recently demonstrated at the Heidelberg Ion-Beam Therapy Center (HIT), with the first patient treatment in 2021 [2], [5], [7]. Despite growing interest in helium ions, wider experience with dosimetric equipment and guidelines remains limited. Absorbed dose calibration for helium ions relies on extensions of the IAEA TRS 398 Rev1 formalism [10]. Accurate correction for stopping-power ratios, ion recombination, and beam quality is essential, particularly in the presence of range-modifying devices.

The same holds for accurate physical and biological modeling for treatment planning. While existing phenomenological and biomechanical models were explored [11], [12], further radiobiological experiments are required in scanned helium ion beams. Additionally, detailed modeling of fragmentation spectra has emphasized the limitations of analytical pencil-beam algorithms and the importance of Monte Carlo (MC)-based reference calculations [13], [14].

The aim of this study was to characterize the physical properties of a helium pencil beamline for research and future clinical application, complementing existing literature on proton and carbon ion commissioning [9], [15], [16], [17], [18]. It comprises accelerator commissioning, covering a wide energy range of 54.6 to 402.8 MeV/u, and the acquisition of beam modeling data for the clinically relevant energy range (65.4 to 220.8 MeV/u). Lower energies are relevant for the treatment of superficial tumors, such as ocular lesions, and for preclinical applications [19], [20], [21], whereas higher energies are promising for helium ion imaging [22], [23], [24]. A commercial treatment planning system (TPS), with an analytical dose computation algorithm for helium ions, was validated against measurements and benchmarked against independent MC simulations.

## 2 Materials and methods

### 2.1 Accelerator, beam delivery infrastructure and commissioning

All measurements were performed at the MedAustron Ion Therapy Center (Wiener Neustadt, Austria), a synchrotron-based facility providing proton and carbon ion therapy and helium ions for research [25], [26], [27]. The beam delivery system (BDS) includes a ripple filter to modulate the energy spread [8]. In addition, a polymethylmethacrylate range shifter with a water equivalent thickness of 35 mm is available. The full accelerator energy range covered 54.6 to 402.8 MeV/u [25], while only energies corresponding to water-equivalent ranges of 30 to 300 mm were considered clinically relevant (65.4 to 220.8 MeV/u).

The synchrotron is capable of providing different spill lengths of 1, 5, and 10 s [25] (2.4 s spill pause in all settings), which were systematically investigated.

The work towards helium ion beam availability proceeded in three phases: (*i*) an *accelerator tuning phase* covering the full energy range, optimizing synchrotron extraction and beam transport for stable scanned beam delivery; (*ii*) a *commissioning phase*, measuring percentage depth dose curves (PDDs), beam optics, and absolute dose across the full energy range to characterize the beam for both research and clinical use; and (*iii*) a *beam model creation and validation phase*, restricted to the clinical energy range, generating and validating a dedicated helium beam model in the TPS.

### 2.2 Dosimetric instrumentation

Beam monitoring and control was performed with the BDS, a set of independent monitoring chambers, located in the nozzle and calibrated following Osorio et al. [28].

PDD measurements and range verification were performed with a PeakFinder (PKF) water column system (PTW, Germany) with a range of 14 to 340 mm [29].

Beam optics (spot size, position, symmetry, and intraspill variation) were measured using the Lynx scintillation detector (IBA Dosimetry, Germany) and evaluated with an in-house developed python code [30]. Reference dose measurements and beam-monitor calibration were carried out with a calibrated PTW-34001-Roos ionization chamber in a stationary water phantom PTW-T41023. Three-dimensional dose verification in a spread-out Bragg peak (SOBP) was performed in a PTW-MP3/PL water phantom using an array of 24 PTW-31015-PinPoint ionization chambers positioned in predefined geometries to capture a grid of dose points in depth and lateral direction [31]. Additional reference dose measurements were performed mid-SOBP with a PTW-30013-Farmer ionization chamber, both in water and in a heterogeneous CIRS 731-HN anthropomorphic head phantom (CIRS, USA), which contains tissue-equivalent materials representing bone and soft tissue.

Supplementary Table S2 summarizes detector systems, measurement purpose, and tolerance criteria, based on clinical requirements established for proton and carbon ion commissioning.

### 2.3 Range measurements

Range verification was performed for beamline commissioning and beam model input covering the full available energy range, for three spill lengths. For energies corresponding to water-equivalent ranges exceeding 340 mm, 20 to 40 cm slabs of RW3 (PTW, Germany), a polystyrene-based solid water-equivalent phantom material (density 1.045 g cm^-3^), were placed upstream of the PKF.

The range was defined as the distal 80 % dose point of the Bragg peak maximum ($R_{80}$). Measured ranges were compared to the nominal range values from the energy tables established during accelerator tuning (Section 2.6) using a tolerance criterion of ±0.3 mm.

### 2.4 Beam optics

Beam optics characterization (spot position, size, and symmetry) was performed for all spill lengths and for representative energies spanning the full energy range using the Lynx detector (Section 2.2). A predefined 3 × 3 spot map with 10 cm spacing was recorded at isocenter to surface distances (ISDs) of -20, 0, 30, 40, 50, and 58 cm, where ISD0 corresponds to an air gap of 66.1 cm and positive values denote moving the isocenter closer to the nozzle. Spot positions were compared to nominal coordinates. Spot sizes were quantified as full width at half maximum (FWHM) in both lateral directions (i.e. x and y), and symmetry was defined as the difference between both directions (mm and %).

Intraspill variation of spot position and size was evaluated for five representative energies (65.4, 113.5, 149.5, 189.7, and 220.8 MeV/u) using a spill length of 10 s.

2D homogeneity of a 10 cm × 10 cm field with 2 mm spot spacing was evaluated for eight energies (54.6, 65.4, 113.5, 149.5, 189.7, 220.8, 332.4, and 402.8 MeV/u) and all three spill lengths. Homogeneity H was defined as:

(1) $$H\left[\text{\%}\right]=\frac{I_{\text{max}}-I_{\text{min}}}{I_{\text{max}}+I_{\text{min}}}\cdot 100,$$

where $I_{\text{max}}$ and $I_{\text{min}}$ are the maximum and minimum pixel intensities, proportional to the locally deposited dose, within the beam field. The 2D homogeneity was evaluated within a field size defined as 50 % beam width excluding the lateral penumbra on both sides (80 to 20 % lateral fall-off).

### 2.5 Beam monitor calibration

The PTW-34001-Roos chamber was cross calibrated against a reference PTW-30013-Farmer type (calibrated at a standard laboratory) according to TRS 398 Rev1 [10] prior measurements. It was positioned with its effective point of measurement at 14 mm depth in water in the PTW-MP3/PL water phantom. Measurements were repeated three times for 25 energies over the full energy range using a spill length of 5 s. Note that these measurements were used for beam model creation.

As described by Palmans and Vatnitsky [32], the energy-dependent beam-monitor calibration function K(E) is defined as:

(2) $$K\left(E\right)=\frac{D_{w,Q}}{S_{E}\left(z\right)\mathrm{MU}}\Delta x\Delta y,$$

where $S_{E}\left(z\right)$ corresponds to the mass stopping power of the helium ions at measurement depth z and a spot spacing of ΔxΔy= 4 mm^2^.

### 2.6 Monte Carlo simulation

MC simulations were performed using GATE 10 with Geant4.11.03 [33]. The fully modeled MedAustron nozzle, previously implemented in GATE v9 [34], [35], was ported to OpenGATE 10 [34]. Simulation parameters are summarized in Supplementary Table S3.

MC simulations served two purposes: (*i*) during accelerator tuning, to support establishment of the energy–range relationship, and (*ii*) after commissioning, as an independent reference for range and beam-optics verification. During tuning, simulations were used to generate an energy list covering ranges of 14 to 300 mm in water in 1 mm steps and additional higher energies up to 402.8 MeV/u (346 energies total) (Supplementary Table S1). Measured ranges of 15 energies were modeled to obtain an initial range-energy model and used to refine accelerator settings. The final energy tables were validated during commissioning with PKF measurements (Section 2.3). After commissioning and acceptance, measured range and beam-optics data were used to generate a MC beam model following published methods [34], providing an independent benchmark.

### 2.7 Beam model creation and verification

Planning and dose computation was performed in a preliminary version of the TPS RayStation v2026 (RaySearch Laboratories, Sweden) using the analytical pencil-beam dose engine version 7.5. Compared to previous versions, this included an improved air-gap model, which combines an adjusted pencil-beam halo width, reproducing the lateral dose horns near the field edges, with range shifter-specific calibration factors that scale the calculated dose as a function of the air gap.

In line with beam model requirements for carbon ions and common guidelines [36], [37], measurements of PDD curves, spot profiles, and absolute dose measurements (Sections 2.3, 2.4, 2.5) were used for beam model creation for the clinical energy range.

For 2D validation of the dose, monoenergetic plans were employed and measured with the PTW-34001-Roos chamber (Section 2.2), averaged over three repeated measurements. The Roos chamber was represented in the TPS by a delineated cylindrical volume, and the mean calculated dose within it was compared with the measurements.

For 3D validation, cube-shaped volumes with a prescribed uniform physical dose of 2 Gy were created with varying sizes and depths [38]. Investigated target volumes with dimensions 3 × 3 × 3 cm${}^{3}$ at 5 cm and 6 cm depth, 4 × 4 × 4 cm${}^{3}$ at 13 cm, 5 × 5 × 5 cm${}^{3}$ at 10 cm and 21.8 cm, and 8 × 8 × 8 cm${}^{3}$ at 15 cm were placed in the water phantom for ISD0. Except for the 8 × 8 × 8 cm${}^{3}$ cube, measurements were repeated at ISD30. Measurements of shallow depths (i.e., 3 × 3 × 3 cm${}^{3}$ at 5 cm depth) were performed with a range shifter at ISD0 and ISD30. Plans were created using a hexagonal spot pattern with a spot interval/range spacing of 0.5 cm/0.2 cm without a range shifter and 0.4 cm/0.5 cm with a range shifter. The dose calculation grid size was set to 2 × 2 × 2 mm${}^{3}$ and all plans were irradiated with a spill length of 1 s. The 24 PinPoint chamber array (Section 2.2) was positioned at predefined depths inside the SOBP.

For each plan, a 3D gamma-index evaluation was performed using global normalization to 2 Gy and with criteria of 3 %/1.5 mm and 5 %/1.5 mm. Voxels below 10 % of maximum dose were excluded. The gamma pass rate was reported as the fraction of evaluated points fulfilling these criteria.

Additionally, validation measurements were performed in the CIRS 731-HN head phantom described in Section 2.2. A cylindrical target volume (r=4cm, h=5cm) was positioned at 7 cm depth and irradiated with a single lateral beam traversing the skull-bone-equivalent material. A uniform physical dose of 2 Gy was prescribed, and plans were delivered at ISD0 and ISD30, both with and without range shifter and for isotropic dose grids of 0.3, 0.2, and 0.1 cm, using the same spot interval and range spacing as for the homogeneous 3D validation. Dose was measured mid-SOBP with the PTW-30013-Farmer chamber positioned inside the phantom and compared to the TPS-predicted dose at the measurement position.

### 2.8 Uncertainty estimations

Uncertainty estimation of dose measurements followed Lechner & Palmans [39]. For the PinPoint chambers, the repeatability term is reported as a range across all 24 detectors. Standard laboratory uncertainties and TRS 398 Rev1 contributions were included [10].

## 3 Results

The combined uncertainty of the PTW-30013-Farmer and the PTW-34001-Roos was 2.5 % and for the PTW-31015-PinPoint up to 2.7 % (Table 1).

**Fig. 1:**
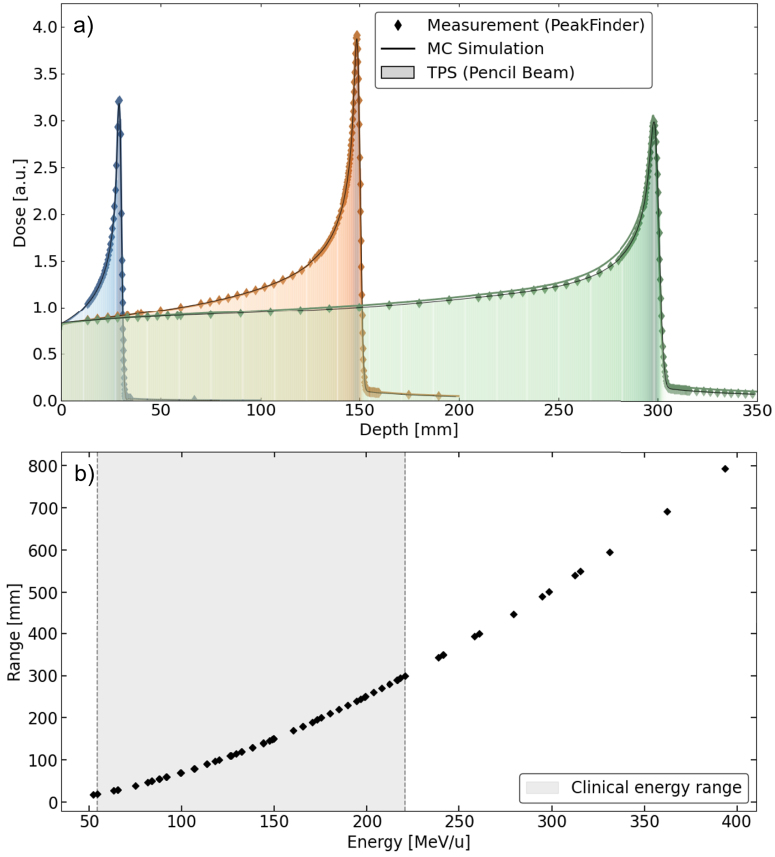
(a) Normalized PDD curves for 65.4, 149.5, and 220.8 MeV/u helium ion beams obtained from MC dose-to-water simulations, PKF measurements, and TPS calculations (1 s spill). Curves were normalized at 14 mm depth and truncated beyond the distal fall-off for visualization. (b) Energy-range relationship for the energy range of 54.6 to 393.4 MeV/u measured with the PKF.

The maximum deviation between measured and nominal range values (Supplementary Table S1) was -0.1 mm. The deviations were randomly distributed across all energies, resulting in a mean absolute deviation of (0.06 ± 0.06) mm. These results demonstrate a stable beam-energy calibration and are consistent with the independent MC-based range validation (Fig. 1).

**Table 1:** Uncertainty budget of dose measurements with the PTW 30013 Farmer, PTW 34001 Roos and the PTW 31015 PinPoint ionization chamber in helium ions. All reported uncertainties are expressed with a coverage factor k=1. Uncertainty No. 3 and 4 for all detectors are based on values provided in the TRS 398 Rev1 [10].

No.	Quantity or standard	Uncert.	Prob. density	Rel.
	source of uncertainty	type	funct.	uncert. [%]
PTW-30013-Farmer				

1	Repeatability, $M_{Q}$	A	Normal	0.2
2	$N_{D,w}$ 60-Co Standardlaboratory	B	Normal	0.7
3	Correction for influence quantities $k_{i}$	B	Normal	0.3
4	Beam quality correction, $k_{Q}$	B	Normal	2.4

**Combined uncertainty - PTW-30013-Farmer**				2.5

PTW-34001-Roos				

1	Repeatability, $M_{Q}$	A	Normal	0.2
2	$N_{D,w}$ 60-Co Standardlaboratory (Reference chamber)	B	Normal	0.7
3	Correction for influence quantities $k_{i}$	B	Normal	0.3
4	Beam quality correction, $k_{Q}$	B	Normal	2.4
5	Cross calibration (PTW-30013-Farmer)	B	Normal	0.2

**Combined uncertainty - PTW-34001-Roos**				2.5

PTW-31015-PinPoint				

1	Repeatability, $M_{Q}$	A	Normal	0.2
2	$N_{D,w}$ 60-Co Standardlaboratory (Reference chamber)	B	Normal	0.7
3	Correction for influence quantities $k_{i}$	B	Normal	0.3
4	Beam quality correction, $k_{Q}$	B	Normal	2.4
5	Cross calibration (PTW-31015-PinPoint)	B	Normal	0.15–0.83

**Combined uncertainty - PTW-31015-PinPoint**				2.5–2.7

**Fig. 2:**
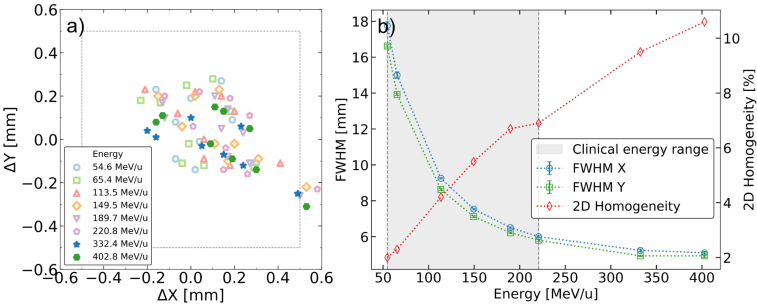
(a) Spot position differences for the predefined 3 × 3 raster (1 s spill) over the full energy range. The black dashed square indicates the ±0.5 mm tolerance window. (b) Energy dependence of lateral FWHM in x and y (mean ± SD) and 2D homogeneity over the full energy range.

Spot FWHM decreased with increasing energy from 17.8 mm at 54.6 MeV/u to 4.8 mm at 402.8 MeV/u. From three measurements, a repeatability uncertainty of ±0.2 mm was obtained. Across all spill lengths, spot size differences for the same energies remained below 1.7 %. MC-simulated spot sizes (transverse FWHM) agreed well with Lynx measurements: differences remained within 0.5 mm across all evaluated energies and isocenter distances, with a mean absolute deviation of 0.1 mm. Apart from three single spots, all measured spot positions were inside the tolerance window of 0.5 mm for all energies and spill lengths (Fig. 2a).

Across all spill lengths, the spot symmetry showed a maximum deviation of 1.4 mm corresponding to 12.8 %, while the mean symmetry deviation was (0.6 ± 0.4) mm corresponding to (7.0 ± 2.8) %. The lateral FWHM in x and y exhibited a monotonic decrease with energy (Fig. 2b).

Intraspill analysis (10 s spill) showed high temporal stability, with spot-position variations ≤0.2 mm and relative spot size variations ≤5.4 %. Across all evaluated energies, the mean intraspill spot-position variation was (0.14 ± 0.05) mm (horizontally) and (0.13 ± 0.08) mm (vertically). Relative spot size variations averaged to (4.0 ± 1.0) % (horizontally) and (4.5 ± 0.9) % (vertically). Slightly larger spot-position variations at 113.5 to 149.5 MeV/u and slightly higher spot size variation at 220.8 MeV/u were observed.

2D homogeneity (Eq. (1)) decreased from 2 % at 54.6 MeV/u to 12 % at 402.8 MeV/u as the spot spacing remained constant while the spot size decreased with energy (Fig. 2b).

**Fig. 3:**
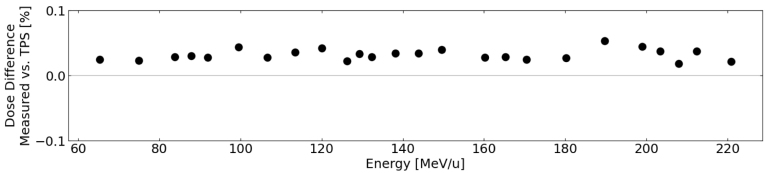
Measured absolute dose compared to TPS values at a reference depth of 14 mm for 65.4 to 220.8 MeV/u.

For 2D dose verification, measurements agreed with TPS values within 0.1 % for all investigated energies (Fig. 3).

**Fig. 4:**
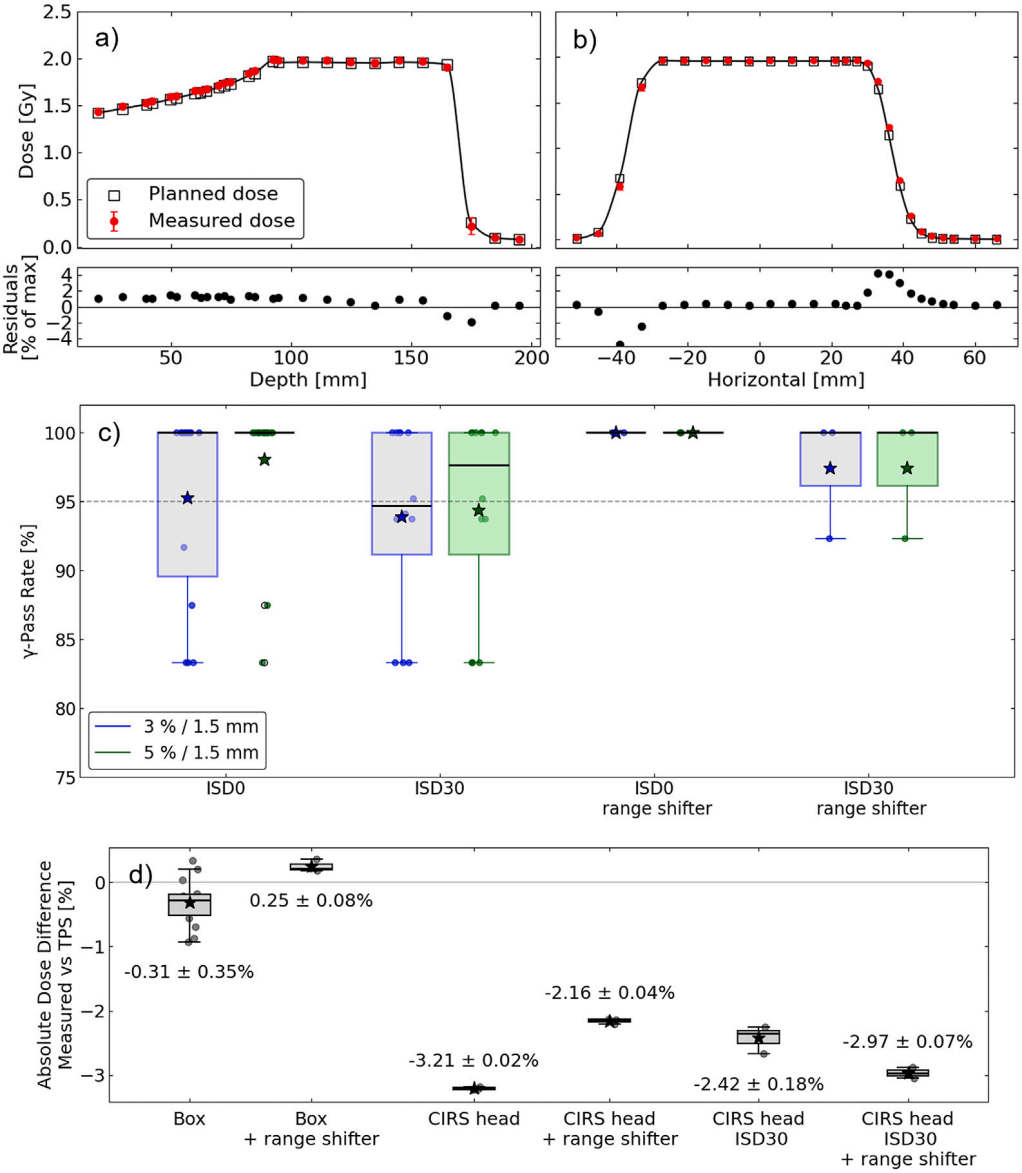
Dose validation results. (a) PDD and (b) lateral profile for a representative cubic volume of 4 × 4 × 4 cm${}^{3}$; residuals (bottom row) are given as % of maximum dose. (c) γ-pass rates for all evaluated cube volumes for 3 %/1.5 mm (blue) and 5 %/1.5 mm (green), grouped by ISD0/ISD30 and range shifter use; the dashed line indicates 95 %. (d) Absolute dose differences at isocenter between measured and TPS dose for each cube and for the CIRS head phantom for all configurations.

For 3D verification, the depth- and lateral profile extracted from the TPS dose distributions showed good agreement with measurements, as illustrated for the representative 4 × 4 × 4 cm${}^{3}$ geometry in Fig. 4a and b. The planned SOBP shape, distal fall-off, and dose plateau were reproduced, and for all cube sizes and depths, deviations between measured and calculated dose values remained within 5 %. Larger deviations (up to 12 %) were observed for shallow fields using a range shifter and in dose gradient regions around 0.1 Gy/mm.

At 5 %/1.5 mm, median gamma pass rates were 100 % (ISD0) and ≥97.6 % (ISD30) without range shifter and 100 % with range shifter at both isocenter distances (min. ≥92.3 %); at 3 %/1.5 mm, 100 % (ISD0) and 94.7 % (ISD30) without range shifter and 100 % with range shifter. Reduced values (down to 83.3 %) were confined to lateral profiles, depth profiles remaining ≥94 % (Fig. 4c).

Mean absolute dose measurements in the SOBP cubes agreed with the TPS. They indicate a small systematic underestimation of (-0.31 ± 0.35) % without range shifter, reduced to (0.25 ± 0.08) % with range shifter, relative to the TPS. These results were consistent with the PinPoint array measurements (Fig. 4d). For the CIRS head phantom, mean absolute dose deviations at ISD0 were (-3.21 ± 0.02) % and (-2.16 ± 0.04) % and at ISD30 (-2.42 ± 0.18) % and (-2.97 ± 0.07) % without and with range shifter, respectively (Fig. 4d).

Variations in isotropic dose grid resolution (0.3, 0.2, and 0.1 cm) had only a minor impact on the observed absolute dose deviations, with differences below 0.1 % without range shifter and below 0.3 % with range shifter (Table 2).

**Table 2:** Absolute dose deviations between measurements and TPS calculations in the CIRS head phantom for isotropic dose grid resolutions of 0.3, 0.2, and 0.1 cm, evaluated with and without range shifter.

Dose grid resolution	Dose difference [%]	
	Without range shifter	With range shifter
0.1 cm	−3.29±0.05	−2.42±0.01
0.2 cm	−3.21±0.02	−2.16±0.04
0.3 cm	−3.28±0.06	−2.36±0.05

## 4 Discussion

The results demonstrated a stable, reproducible, and well- characterized helium ion beam, meeting the requirements for dosimetric and biologically driven research and for future clinical applications.

Across spill lengths of 1, 5, and 10 s, beam characteristics remained consistent, comparable to protons and carbon ions. The FWHM was effectively independent of spill length, indicating that the extraction time structure did not measurably affect the lateral phase space. Spot sizes showed the expected decrease with increasing energy [40]. Intraspill stability remained high, below 0.2 mm, and therefore comparable to proton and carbon commissioning experience [41], [42]. Beam-monitor calibration provides a spot positioning resolution of 0.3 mm [28], while scanning-magnet response, intensity-ripple suppression, and interlock timing characterized for the BDS [43] apply identically to helium.

The measured beam characteristics were consistent with published helium commissioning results from HIT, despite differences in accelerator and delivery infrastructure [5], [44]. Low intraspill variations were consistent with the reported stability metrics, supporting the reproducibility of helium beam properties across different synchrotron-based facilities. The demonstrated stable output at a high dose rate (i.e. 1 s spill length) provides a unique setting for dosimetric characterization of detectors in helium beams, as well as for calorimetric measurements [45], [46].

Beam model commissioning demonstrated excellent agreement for standard geometries. For plans including the range shifter, the improved air-gap model reduced the absolute dose deviation in water to within 0.25 %. The remaining inaccuracies were confined to the heterogeneous head phantom and steep-gradient regions, consistent with known limitations of pencil-beam algorithms under increased scattering [47], [48], [49], [50].

MC-simulated PDD curves agreed well with measurements with differences observed at higher energies, where MC predicted slightly lower entrance dose and slightly higher distal-tail dose than PKF measurements, likely due to inaccuracies of inelastic physics process modeling.

The up to 3 % larger dose deviation in the heterogeneous head phantom compared to water was consistent with findings for protons and carbon ions at MedAustron [38], [41], [51], suggesting limitations of the pencil-beam algorithm in modeling lateral scattering at bone–tissue interfaces. Future clinical helium ion patient-specific quality assurance would follow existing proton and carbon ion workflows, using the 24 PinPoint chamber array [31] and independent dose calculation.

The isotropic dose grid resolution had only a minor impact on the absolute dose deviations, indicating that the observed discrepancies are dominated by limitations of the pencil-beam algorithm rather than dose grid sampling effects. In addition, assessing the sensitivity of helium ions to motion-induced interplay effects might be necessary, depending on the tumor site [52], [53], [54].

The ion recombination and polarity correction factors used in this work were adopted from previous internal commissioning experience with protons and carbon ions [9], [28], [29], which built upon earlier studies [55], [56], [57]. However, the mechanism of ion recombination is complex and depends on various parameters, including LET (initial recombination) and dose rate (volume recombination) [58], [59]. While initial recombination becomes increasingly important for high-LET particle beams, volume recombination can no longer be neglected at the high instantaneous dose rates seen in scanned beams, which are two to three orders of magnitude higher than in scattered delivery [56], [57], [58], [60]. For proton beams, ion recombination corrections of up to 0.5 % have been reported for a Markus-type chamber [55], [58]. Polarity corrections have likewise been characterized for plane-parallel chambers in synchrotron-based scanned proton and carbon ion beams [59]. It is recommended in TRS 398 Rev1 [10] to determine chamber-specific $k_{s}$ correction factors by a Jaffé-plot analysis, as this method can also be applied to high-LET beams and was recently extended to scanned particle beams [57], [61]. A more detailed investigation of the ion recombination and polarity correction in helium beams remains the subject of future investigations. Applied $k_{Q}$ values were associated with an uncertainty of 2.4 %. Dedicated values for helium ions with a lower uncertainty were recently published [46].

While this study focused on absorbed dose to water, the commissioned beamline enables future investigations of helium-specific RBE models, multi-ion planning strategies to shape dose and LET distributions [62], [63], [64], helium ion imaging [22], [23], [24], [65], and pre-clinical studies [19], [20], [66].

In conclusion, the commissioned helium beamline met all physical and dosimetric requirements for future clinical application and high-level research projects. Equipment and methods previously applied to proton and carbon ions could successfully be transferred to helium ion commissioning. The validated beam model provides a robust basis for biological modeling, RBE-weighted dose evaluation, and further translational work.

## CRediT authorship contribution statement

**Lukas Martin:** Writing – original draft, Visualization, Methodology, Investigation, Data curation, Conceptualization. **Barbara Knäusl:** Writing – review & editing, Supervision, Methodology, Conceptualization. **Peter Kuess:** Writing – review & editing, Visualization, Methodology, Investigation, Data curation, Conceptualization. **Dietmar Georg:** Writing – review & editing, Resources, Methodology. **Hugo Palmans:** Writing – review & editing, Supervision, Methodology, Investigation, Conceptualization. **Lorenz Wolf:** Writing – review & editing, Validation, Software, Data curation. **Nadia Gambino:** Validation, Software, Investigation. **Fabio Farinon:** Validation, Software, Investigation. **Andrej Prochazka:** Validation, Software, Investigation. **Mansure Schafasand:** Visualization, Software, Investigation, Data curation. **Lars Glimelius:** Software, Formal analysis. **Walter Ikegami Andersson:** Software, Formal analysis. **Daniel Simon Colomar:** Software, Formal analysis. **Antonio Carlino:** Visualization, Methodology, Conceptualization. **Markus Stock:** Writing – review & editing, Supervision, Resources, Methodology, Conceptualization. **Hermann Fuchs:** Writing – review & editing, Supervision, Software, Methodology, Investigation, Conceptualization.

## Declaration of competing interest

Barbara Knäusl is an Editor-in-Chief and Hugo Palmans is a co-Guest Editor for this journal and were not involved in the editorial review or the decision to publish this article.

The authors declare the following financial interests/personal relationships which may be considered as potential competing interests:

Given her role as Editor-in-Chief, Barbara Knäusl had no involvement in the peer-review of this article and had no access to information regarding its peer-review. Full responsibility for the editorial process for this article was delegated to another journal editor.

The MedAustron Ion Therapy Center and the Medical University of Vienna both hold research contracts with RaySearch Laboratories.

## Appendix A Supplementary data

MMC S1 Supplementary tables: range–energy measurements, detector systems and tolerance criteria, and Monte Carlo parameters. MMC S1
