# Supplementary material for: Helium pencil beam commissioning and beam modeling
Source: Phys Imaging Radiat Oncol. 2026 Jul 26;40:101046. doi: 10.1016/j.phro.2026.101046 (PMC13449418; doi:10.1016/j.phro.2026.101046)
Supplement: Supplementary file 1 [file mmc1.pdf]

Supplementary Material

Helium pencil beam commissioning and beam modeling

Table S1: Range measurements (R_{80}) with the PeakFinder at 80 % of the dose maximum after the Bragg peak for the potential clinical energy range.

**Energy [MeV/u]**	**R_{80} [mm]**
65.4	29.96
70.3	34.94
75.0	39.95
79.5	45.00
83.8	50.02
91.9	60.02
99.5	70.06
106.5	79.96
113.5	90.07
120.0	100.01
126.3	110.04
132.4	120.08
138.2	129.97
143.9	139.96
149.5	150.07
154.9	160.05
160.2	170.07
165.3	179.95
170.4	190.03
175.4	200.09
180.2	209.97
185.0	219.98
189.7	229.98
194.2	240.12
198.9	249.98
203.4	259.96
207.9	270.10
212.3	280.14
216.6	290.14
220.8	299.94

Table S2: Summary of measured beam parameters, detector systems, purposes, and acceptance criteria during helium beam commissioning.

Beam Parameter	Detector System	Purpose	Tolerance Criterion
Range (R_{80})	PeakFinder (PTW)	Energy–range calibration and delivery accuracy	±0.3 mm deviation
Lateral spot size (FWHM)	Lynx (IBA)	Beam optics characterization	≤5 mm at isocentre
Spot position accuracy	Lynx (IBA)	Verification of geometric beam delivery accuracy	≤0.5 mm deviation
Depth–dose distribution	24 PP IC (PTW)	Verification of TPS calculated 3D dose distribution	≤5% dose deviation
Lateral dose profile	24 PP IC (PTW)	Verification of lateral halo and SOBP uniformity	≤5% dose deviation
Absolute dose to water	Farmer IC (PTW 30013)	TPS validation	≤2.6% (no range shifter), ≤5.5% (range shifter)
Intraspill stability	Lynx (IBA)	Assessment of temporal beam stability	≤6% change in FWHM

Table S3: Parameters employed for the Monte Carlo simulation and optimization.

Parameter	Medium / Category	Value
Physics list	helium ions	ShieldingLIQMD_HP
Maximum production & tracking cuts	air	10 cm
	water (G4_Water)	0.1 mm
	nozzle materials	10 mm
Statistical uncertainty	—	< 2%
Bin size	integrated depth–dose curve	0.1 mm
	lateral profiles	0.1 mm^2
